# Uncovering secondary metabolite evolution and biosynthesis using gene cluster networks and genetic dereplication

**DOI:** 10.1038/s41598-018-36561-3

**Published:** 2018-12-18

**Authors:** Sebastian Theobald, Tammi C. Vesth, Jakob Kræmmer Rendsvig, Kristian Fog Nielsen, Robert Riley, Lucas Magalhães de Abreu, Asaf Salamov, Jens Christian Frisvad, Thomas Ostenfeld Larsen, Mikael Rørdam Andersen, Jakob Blæsbjerg Hoof

**Affiliations:** 1 Department of Biotechnology and Biomedicine, Technical University of Denmark, DK-2800 Kongens Lyngby, Denmark; 2 Department of Energy Joint Genome Institute, Walnut Creek, CA USA; 3 Department of Plant Pathology, Federal University of Viçosa, Viçosa, Brazil; 4 Present Address: The Novo Nordisk Foundation for Biosustainability, Technical University of Denmark, DK-2800 Kongens Lyngby, Denmark; 5 Present Address: Chr. Hansen Holding A/S, DK-2970 Hoersholm, Denmark; 6 Present Address: Amyris, Inc., Emeryville, CA USA

## Abstract

The increased interest in secondary metabolites (SMs) has driven a number of genome sequencing projects to elucidate their biosynthetic pathways. As a result, studies revealed that the number of secondary metabolite gene clusters (SMGCs) greatly outnumbers detected compounds, challenging current methods to dereplicate and categorize this amount of gene clusters on a larger scale. Here, we present an automated workflow for the genetic dereplication and analysis of secondary metabolism genes in fungi. Focusing on the secondary metabolite rich genus *Aspergillus*, we categorize SMGCs across genomes into SMGC families using network analysis. Our method elucidates the diversity and dynamics of secondary metabolism in section *Nigri*, showing that SMGC diversity within the section has the same magnitude as within the genus. Using our genome analysis we were able to predict the gene cluster responsible for biosynthesis of malformin, a potentiator of anti-cancer drugs, in 18 strains. To proof the general validity of our predictions, we developed genetic engineering tools in *Aspergillus brasiliensis* and subsequently verified the genes for biosynthesis of malformin.

## Introduction

The genus *Aspergillus* is one of the best studied fungal genera, with important species in the industrial, food and medical sector as well as in basic research. Its diverse repertoire of bioactive secondary metabolites (SMs) e.g. anti-cancer compound enhancing malformins, cholesterol-lowering statins, and the toxic aflatoxins have been detected in numerous analytical studies^1^ — with many SMs applied primarily in the medical industry^2^.

SMs are synthesized by different classes of enzymes. In fungi, these are polyketide synthases (PKSs), non-ribosomal peptide synthetases (NRPSs), terpene cyclases (TCs), dimethylallyl tryptophan synthases (DMATSs), enzymes consisting of a smaller subset of modules (PKS-Likes, NRPS-Likes), and fusions of PKS and NRPS (PKS-NRPS/NRPS-PKS hybrids). These enzymes produce a SM backbone which is further modified by tailoring enzymes. The collective of enzymes necessary for production of a SM is encoded by a gene cluster. SMs can also be ribosomally synthesized and posttranslationally modified peptides (RiPPs)^3,4^ which have precursor peptides located in a gene cluster.

NRPSs constitute a major group of secondary metabolite enzymes and can utilize L-amino acids, as well as non-proteogenic amino acids as their substrate^5^, creating a diverse portfolio of compounds. Domains inside NRPSs are adenylation domains (A) for loading of amino acids, thiolation (T) domains for peptide chain transfer, condensation domains (C) for peptide bond formation, and epimerisation domains (E) to change the chirality of their proximate amino acid. Most NRPSs investigated show a colinearity rule, meaning they are assembled as modules in the order ATC. *Euascomycete* specific groups of NRPSs show substantial gain and loss of domains, further emphasizing the role of this enzyme class in chemical evolution of fungi^6^. Understanding these dynamics and describing the diversity of NRPSs - and other secondary metabolites - throughout the genus *Aspergillus* will lead the way for new pharmaceutical drugs.

Prediction pipelines such as SMURF^7^ and antiSMASH^8^ facilitate the mining of genomic sequences for secondary metabolite gene clusters (SMGCs). To efficiently analyse these large datasets across several organisms, genome neighbourhood networks have been used previously in bacteria to predict new gene clusters and ease strain prioritization for polyketides of interest^9–11^. However, these approaches are either limited on a narrow class of SMGCs, only use conserved domains to infer gene cluster similarity, or they require manual sorting of SMGCs.

In this study, we made a thorough analysis of SMGC dynamics throughout section *Nigri* to investigate species similarities on the SMGC content level and genetically dereplicate gene clusters using secondary metabolite gene cluster networks. In particular, we provide details on the pipeline we have generated for generating families of SMGCs and used the pipeline to find gene clusters for analogous compounds in newly sequenced genomes.

We used this pipeline to describe the dynamics and diversity of annotated and non-annotated SMGCs of 32 genomes (26 species) of the SM-rich *Aspergillus* section *Nigri*^1^ and five reference species. Section *Nigri* is particularly interesting, as it is both rich in secondary metabolism and contains several species relevant for biotechnological applications as cell factories^12,13^ Identifying homologous gene clusters on the isolate, clade and section level enabled us to define groups with similar SMGC content inside section *Nigri*.

As an extension of our approach, we demonstrate the use of SMGC families together with information on metabolite profiles to mine for the gene cluster responsible for malformin biosynthesis. Malformins, a major group of compounds abundant in section *Nigri*^14^, show anti-tobacco mosaic virus activity^15^ and act as potentiator of anti-cancer drugs in mouse and human colon carcinoma cells^16^. Identifying the SMGC responsible for malformin biosynthesis will allow for optimization of native as well as heterologous gene expression. Our approach successfully predicts the malformin gene cluster in multiple *Aspergillus* species, unveiling the feasibility of performing large scale dereplication of homologous gene clusters using collections of genome-sequenced strains.

## Results

### Creating families of secondary metabolite gene clusters

In order to describe the SMGC diversity of section *Nigri*, we analyzed 32 *Aspergillus* genomes of this section as an extension of our previously published work^12^. Five reference species: the industrially relevant *A. oryzae*, pathogenic *A. flavus* and *A. fumigatus*, the model organism *A. nidulans*, and the related fungus *Penicillium chrysogenum*, were added to investigate their similarity to species of section *Nigri*. Aspergilli are known to produce similar SMs across species^1,17,18^, thus including these species would ensure to relate SMGC content to phylogeny and potentially reveal homologous gene clusters. The first genomic analysis of section *Nigri* showed great abundance and diversity of SMGC^12^.

**Figure 1:**
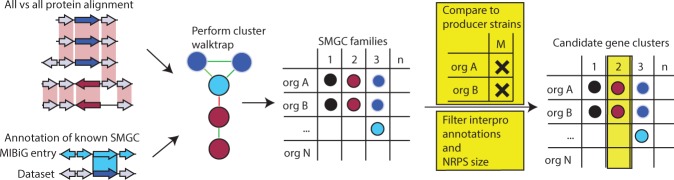
Workflow of the bioinformatic pipeline. Prior to data analysis gene annotation, InterPro and SMURF data are combined. SMGC are compared using protein BLAST of cluster members and percent identity values of alignments are aggregated to cluster similarity scores and used to create a gene cluster network. Additionally, known gene clusters from the MIBiG database are annotated in the dataset by identifying an exact match. Random walk clustering is performed using the cluster walktrap function^52^ of igraph^51^ on the network to obtain families of SMGC. To identify candidate SMGC for metabolites of interest, lists of metabolite producing organisms are compared to lists of organisms containing SMGCs of the same family. Candidate SMGC families are filtered by interpro annotations and e.g. NRPS size.

Using a pipeline we have built for dereplicating SMGCs by sorting them into families (see outline in Fig. 1), we detected 2,622 gene clusters and categorized them into 435 families — groups predicted to produce similar compounds based on homologous gene clusters — of which 217 only contain one cluster and are therefore unique.

### Comparative genomics reveals secondary metabolite gene cluster diversity on several taxonomic levels in section *Nigri*

**Figure 2:**
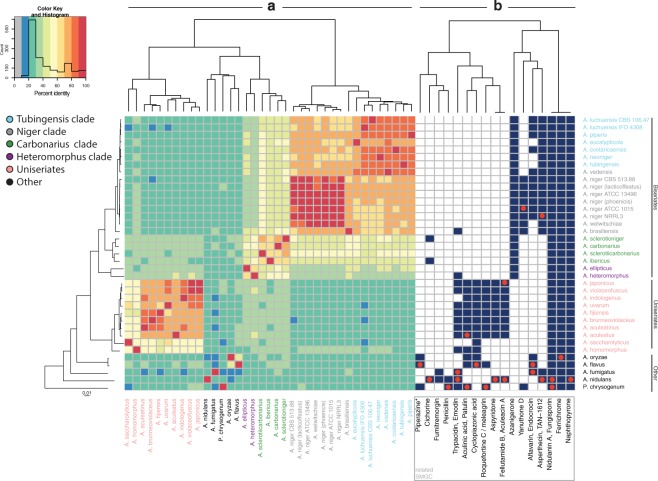
Heatmap of shared SMGC families and gene clusters linked to compounds. This heatmap contains information on phylogeny of used strains, shared SMGC families and metabolite-linked gene clusters based on MIBiG entries. The row dendrogram represents a whole genome phylogeny. The column dendrogram was generated by creating a distance matrix of shared SMGC families by organisms and running hirarchical clustering with euclidean distance (part of the heatmap.2 function). (**a**) Relative amounts of shared SMGC families between species in percent. Here, the presence of SMGC families resulting from our pipeline was compared through all species. Percentage is indicated as color gradient in bins of 10% from grey cells (0–10%, not present in dataset) to red cells (90–100%) as shown by the color key. Additionally, a histogram indicates the abundance of different amounts of shared SMGC families, hence, how many comparisons result in low or high similarity respectively. Species self-comparison always results in values of 100%. The column dendrogram represents a hierarchical clustering of organisms by shared SMGC percent, hence strains clustering together will share a high amount of SMGCs. (**b**) Identification of compound-linked gene clusters based on MIBiG entries. Best hits for MIBiG entries, were identified inside families using protein BLAST (red dot). Aculinic acid and emodin gene clusters were confirmed by sequence identifier. Using a guilt-by-association approach, the whole family of gene clusters is considered to be responsible for the production of a similar metabolite. The heatmap column dendrogram is clustered hierarchically based on presence of compound-linked gene clusters. Dereplicated gene clusters that do not show related gene clusters in other species were removed. 4,4′-piperazine-2,5-diyldimethyl-bis-phenol is abbreviated as piparazine*.

With the establishment of SMGC families in related species, we were interested in whether SMGC content is reflected in species, clade, section and genus circumscription. Differences in SM content have been shown previously for the groups of uniseriates (species with phialides attached directly to the vesicle) and biseriates (species with metulae between phialides and vesicle) inside section *Nigri* (Fig. 2)^1^. Additionally, SMGC content has been shown to be dynamic for the species of this section. Hence, differences in SM production should be the result of different SMGCs present in species.

Comparing all shared SMGCs throughout species in the dataset enables us to highlight groups of species carrying similar SMGCs (Fig. 2); showing a clear distinction between biseriate species, uniseriate species and reference species. Inside these groups, further subgroups can be identified.

The *A. niger* isolates shared 80–100% of SMGC families — pointing out that isolates of the same species can carry a few different SMGCs — although none of them have unique SMGCs. The shared SMGC content among species varied depending on the clade. Species in the *A. niger* and *A. tubingensis* clade share 60–80% — with *A. eucalypticola* showing a distinct SMGC composition inside the clade. Species in the *A. carbonarius* clade share 60–80% of SMGC families. This similarity dropped to 50–60% inside the *A. heteromorphus* clade. Most uniseriates shared at least 70% SMGCs, with the exception of *A. saccharolyticus* and *A. homomorphus* only sharing as few as 40% of their SMGC families with other members of the uniseriates. On a section level, we can show that biseriates and uniseriates (apart from the *A. heteromorphus* clade) each show a SMGC family inter-clade similarity of at least 30%. Comparing the *A. tubingensis* and *A. niger* clade to uniseriates the SMGC similarity is 20–30% — the same as between the section *Nigri* and the reference species. Hence, we can determine that the diversity of secondary metabolites inside section *Nigri* is similar to the diversity seen across the genus as a whole.

From this, it can be inferred that section *Nigri* must have undergone a substantial gain and loss of secondary metabolite genes. In species which show a larger difference in SMGC composition to closely related species — as in the case of *A. eucalypticola* — suggests horizontal gene transfer from outside section *Nigri* or retention of SMGC. Surprisingly, a small amount of SMGCs seem to be retained in the whole genus since we find at least 10% similarity of SMGC families between the species included in the analysis. Additionally, a maximum of 30% shared SMGC families between distantly related species exceeds the SMGC similarity previously anticipated in the genus *Aspergillus*^19^.

In conclusion, we can confirm that the clustering of SMGCs into families reflects the SM distribution of species in analytical studies. The SMGC similarity over large phylogenetic distances suggests analogous pathways in the same family.

### Coupling MIBiG annotation to SMGC families automates genetic dereplication of compounds

With the diversity of SMGCs established through our dataset, we were interested in the presence of gene clusters linked to known compounds through section *Nigri*. With an increasing number of available fungal genome sequences, we see an identification of known compounds by genomic methods as crucial, since laboratory conditions might not reveal the full metabolite profile of a fungus. Furthermore, it may help to avoid experiments re-identifying the same gene cluster in multiple species (similar to the process known as metabolite dereplication in chemical analysis^20^). To achieve this, we used 1461 gene clusters of the Minimum Information about a Biosynthetic Gene cluster (MIBiG) database^21^ to identify known compounds with characterized SMGCs in our SMGC families and determine related compounds. This is of special interest for mycotoxins and compounds with medical applications.

Using protein BLAST^22^, we identified 36 best hits found in our SMGC families for compound-linked gene clusters retrieved from MIBiG. Since SMGC families represent groups of homologous and related gene clusters, we can identify the SMGC family of the hit as a related gene cluster producing a similar compound by using a guilt by association approach. Hence, new genomes can be analyzed and added with information on their secondary metabolite production capabilities. The associated compounds and presence patterns of gene clusters are shown in Fig. 2b.

Of the 36 known gene clusters used to annotate the SMGC families in the dataset, two gene clusters linked to the compounds fungisporin, YWA1 and one gene cluster family linked to the siderophore ferrichrome were found in all species of the dataset (Fig. 2). This illustrates that we can detect homologous gene clusters over the genus.

### SMGCs for highly similar compounds are found in shared SMGC families

As a further validation of the method, and to make sure that the algorithm could sort structurally related compounds into the same families, we checked for the gene clusters producing the structurally related polyketides asperthecin and TAN-1612, a neuropeptide Y antagonist^23^. With the set parameters for calculating the similarity of clusters, these are indeed found in the same SMGC family (Fig. 2).

Of further interest, the gene cluster in *A. nidulans* is producing asperthecin, while the gene clusters predicted in the *A. niger* and *A. tubingensis* clades are likely producing TAN-1612 since the uniform presence in these two clades suggests the gene cluster to be conserved throughout species (Fig. 2b). This highlights further that our method can be used to mine for similar compounds in SMGC families.

### SMGCs for similar clusters can be detected across phylogenetic distance

We further wanted to check the assignment of SMGC families across larger phylogenetic distance. Interestingly, the generated SMGC families (Fig. 2) show a family with section *Nigri* uniseriate members sharing a gene cluster also found in *A. flavus*^24^ and *A. oryzae*^25^ responsible for the production of food contaminant and mycotoxin cyclopiazonic acid (CPA). It is surprising that the gene cluster is also found in *A. saccharolyticus* and *A. heteromorphus* since they differ in their SMGC content from the rather SM homogeneous rest of uniseriates (Fig. 2). Kato *et al*.^26^ highlighted that CPA is produced but converted in *A. oryzae*, so it remains to be answered if the uniseriate species produce CPA or a derivate thereof. This confirms our findings that a number of SMGCs can be shared over large phylogenetic distances and the algorithm can detect these.

### SMGCs for different heteroisoextrolites based on 6-MSA are found in section *Nigri*

Aspergilli in distinct sections are known to produce functionally similar types of secondary metabolites, also called heteroisoextrolites^18^. These heteroisoextrolites are based on analogous biosynthetic pathways which we successfully annotated in gene cluster families.

Using our automated method, we were able to detect SMGCs for heteroisoextrolites that are based on 6-methylsalicylic acid (6-MSA), in particular the antifungal patulin^27,28^ and the antimicrobial yanuthone D^29,30^. Inspection of the family associated to the patulin gene cluster shows nine patulin-like gene clusters in uniseriates and the aculinic acid gene cluster, which is highly similar in genetic content and function to the patulin gene cluster^29^, in *A. aculeatus*. Gene clusters primarily found in the *A. niger* clade, as well as in *Penicillium chrysogenum* are predicted to produce the antifungal 6-MSA-based compound yanuthone^29,31^. The network plot in Fig. S1 shows how the related clusters were divided into families and highlights how SMGC networks can be used to classify related SMGCs.

Furthermore, we could infer candidate gene clusters for secalonic acid, a compound with a wide range of bioactivities^32,33^ produced by uniseriates^17,34^, through association of uniseriate gene clusters with the emodin gene cluster^35^ from *A. nidulans* and the trypacidin gene cluster from *A. fumigatus*^36^.

Previous studies identified the silent azanigerone gene cluster by overexpression of cluster genes in *Aspergillus* niger ATCC 1015^37^. In our analysis, we can identify an azanigerone-like producing gene cluster in biseriates, and *A. homomorphus* — which is uniseriate (Fig. 2). This further highlights our algorithm as an important addition to chemical analysis, since genetic dereplication is able to identify gene clusters over a large set of genomic sequences, even though they may be silent in the hosts under normal conditions.

Furthermore, our analysis can automatically identify related SMGCs over a large set of species. Our analysis also highlights that genetically dereplicated SMGC only constitute a small fraction of the secondary metabolites potentially produced by Aspergilli.

### Mining for gene clusters in SMGC families reveals candidates for the malformin gene cluster in 18 strains

To address the large interest in discovery of novel biosynthetic gene clusters for compounds of interest, we wanted to link SMGC families to compounds of interest. For this, we focused on malformin producing species: *A. niger*, *A. brasiliensis*, and *A. tubingensis*^1^. Malformin is interesting as it is both a potential compound for medical application in cancer treatment^16^ and is produced under laboratory conditions. Genome mining for a producer NRPS however, was not trivial since the mentioned species contain between 15 and 17 NRPS gene clusters^12^.

**Figure 3:**
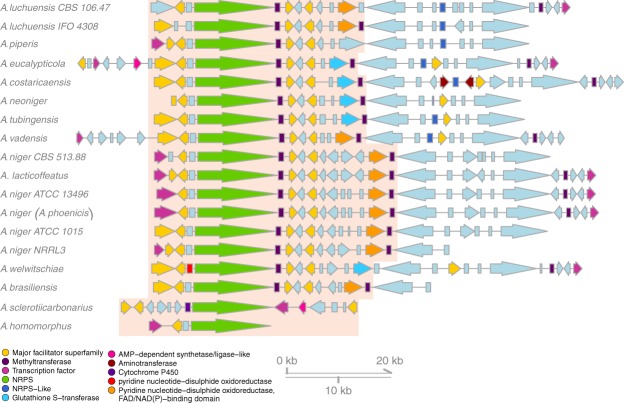
Predicted SMGC family for malformin producing gene clusters. InterPro annotations are indicated by color. The predicted SMGC family contains gene clusters with an NRPS gene of at least 12,000 bp. SMURF predicted gene clusters are shown in full; the predicted malformin gene clusters are highlighted. Tailoring genes code for enzymes like major facilitator superfamily and transcription factors as well as enzymes involved in disulphide bond formation.

First, we searched the output of the pipeline for all analogous NRPS gene clusters present in all producing species. Malformin is a pentapeptide consisting of Val, D-Leu, Ile and two D-Cys amino acids (malformin C). Hence, as a first hypothesis, we assumed the NRPS would consist of five modules with a length of approximately 18,000 bp. However, no such clusters existed in the data. We thus moderated our search to four modules under the hypothesis that one of the modules is iterative (as seen in studies on bacterial sequences^38^), resulting in a minimum size of 12,000 bp. Furthermore, we included the assumption that tailoring enzymes in the cluster should contain disulphide bond-associated enzymes to be able to create the disulphide bond included in malformins. By comparison of the algorithm results to producing species and filtering by the criteria mentioned above, a single candidate gene cluster family was found with matching NRPS size, high level of synteny, and disulphide bond creating enzymes (Fig. 3). In summary, the algorithm allowed us to narrow the search from thousands of SMGCs, to a single candidate.

**Figure 4:**
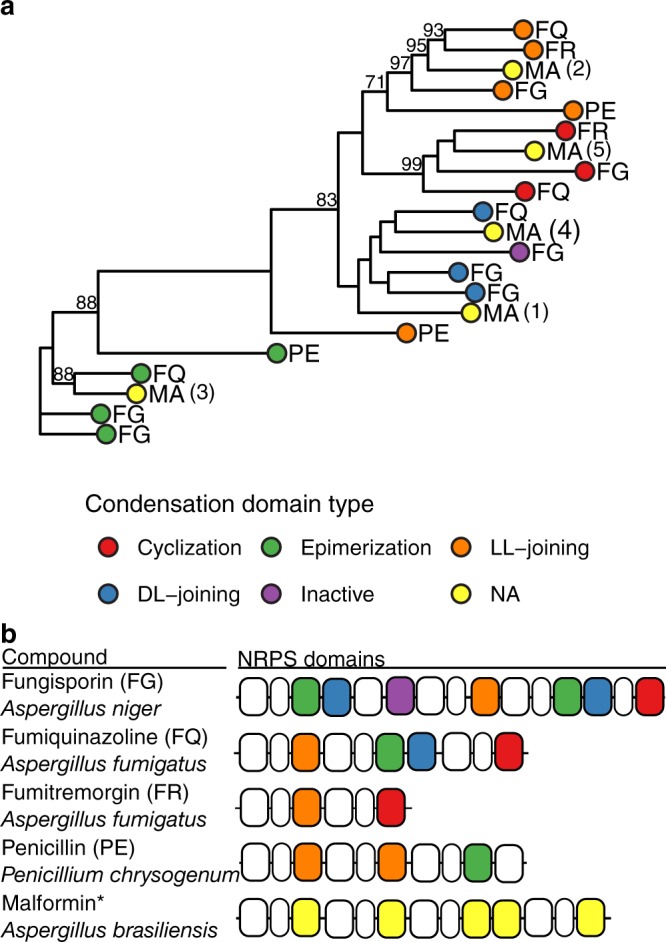
Classification of condensation domains inside the predicted NRPS responsible for malformin synthesis. (**a**) Approximate maximum likelihood phylogeny of condensation domain amino acid sequences. Sequences of condensation domains with known activities from fungisporin (FG), fumiquinazolines (FQ), fumitremorgin (FR) and penicillin (PE) were used to infer activities of condensation domains in the predicted malformin (MA) producing NRPS. The tree was generated from 60% of conserved aligned columns and bootstrapped 1,000 times. Bootstrap values over 70 are shown next to their node. The analysis shows distinct clusters corresponding to functions of condensation domains supported by high bootstrap values. (**b**) Schematic for used NRPS proteins. Condensation domains are highlighted according to their function as depicted in the legend (NA: not available). Adenylation and pcp domains are represented by white cells.

To further confirm the predicted cluster as the best candidate, we created a maximum likelihood phylogeny of NRPS condensation domains with known functions in our dataset (fungisporin/nidulanin A^39–41^, fumiquinazolines^42^, fumitremorgin/brevianamide^43^ and penicillin^44^), including condensation domains of the predicted malformin synthetase MlfA to predict their functions (Fig. 4). According to the amino acid composition of malformin, we expected epimerization, epimerizing D-L joining condensation domains and a cyclizing condensation domain to be present in the synthetase. From branches in the phylogeny, we can predict the functions of the five condensation domains in malformin to be DL-joining (epimerizing subtype), LL-joining, epimerization, DL-joining and cyclizing domain, thus matching the expectations and supporting the identification of the NRPS as involved in malformin production.

The gene cluster family was curated by removing four gene clusters shown in Fig. S2, which only aligned to the extended part of the predicted cluster and not to the NRPS part of the cluster. Extension of gene clusters is an expected behaviour when working with automated annotation of SMGC by SMURF and is easy to identify by synteny analysis using SMGC families generated by genetic dereplication. The SMGC families thus help improve some of the shortcomings of automated SMGC prediction, by giving access to the synteny data across related clusters.

### Genetic and chemical analysis verifies *mlfA* prediction

**Figure 5:**
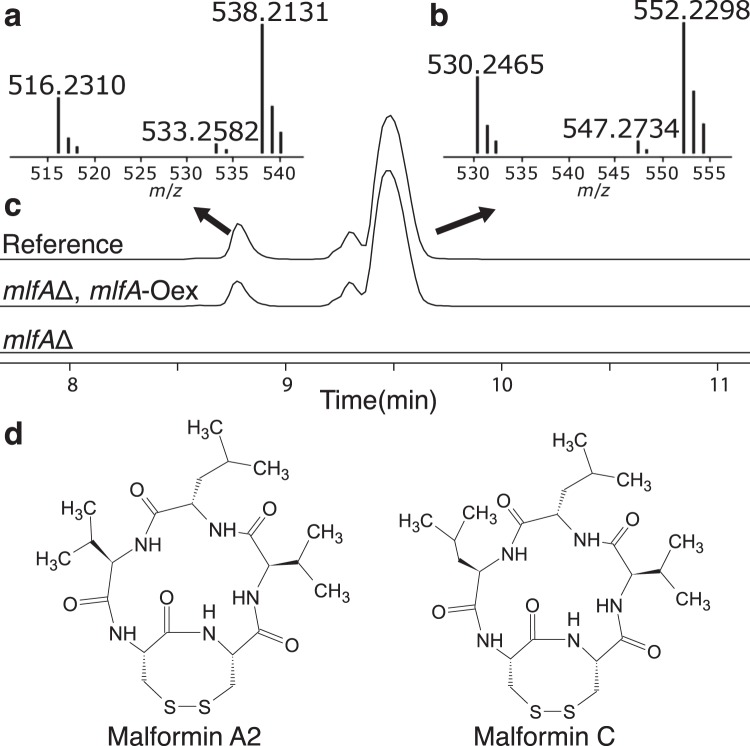
Extracted Ion Chromatograms (EIC) for malformin overexpressing (*mlfA*Δ, *mlfA*-Oex) and malformin knock-out (*mlfA*Δ) strains. (**a** and **b)** show MS spectra of detected adducts [M^+^H]^+^, [M^+^NH4]^+^ and [M^+^Na]^+^ for the peaks displayed in (**c**) showing merged EICs of the six adducts (±0.005 Da) in the reference strain (*akuA* Δ::AFL*pyrG*), *mlfA*Δ, *mlfA*-Oex (*mlfA*Δ IS1::PgdpA-*mlfA*) and *mlfA* deletion strain (*mlfA*Δ). (**a**) reveals the peak at RT 8.9 min contains calc. *m*/*z* 516.2310, 533.2582, 538.2131, corresponding to adducts of low-mass malformins, e.g. A2 (**d**). The two peaks at RT 9.4–9.7 min contain the adducts of high-mass malformins, calc. *m*/*z* 530.2465, 547.2734, 552.2298 (**b**), where the largest peak at RT 9.7 min represents malformin C as determined by comparison to a reference standard of malformin C (**d**). The small peak at RT 9.4 min denotes another of the high-mass malformin (e.g. malformin A1, B1, B3, B4)^72^. In (**c**) the vertical axis displaying MS counts is not shown, however the intensity of the tallest peak is approximately 2 × 10^6^.

To verify the genetic assignment, we first had to develop genetic engineering tools in *A. brasiliensis*. We decided to construct a *pyrG*Δ strain in order to subsequently generate a non-homologous end-joining deficient strain — facilitating efficient gene targeting^45^. We employed a clustered regularly interspaced short palindromic repeats associated endonuclease 9 (CRISPR-Cas9) system^46^ to induce a double-strand break (DSB) in *pyrG* resulting in uridine auxotrophy. Subsequent sequencing of three candidates confirmed that strain 1 had an out-of-frame mutation in the region corresponding to the protospacer via a 16-nucleotide deletion (nucleotides number 45–60, allele name *pyrG1*) within *pyrG*. In this strain, we utilized the CRISPR-Cas9 system to induce a DSB at the *akuA* locus while supplying a repair template in form of a linear gene-targeting substrate for *akuA*. A correct homokaryotic transformant was verified as an *akuA* deletant by diagnostic tissue polymerase chain reaction (PCR) (Fig. S3). The strain, *akuA*Δ::AFL*pyrG*, was screened on 5-fluoroorotic acid (5-FOA) enriched growth medium for loss of AFL*pyrG* by the lack of ability to grow without supplementation of uridine in the medium and diagnostic PCR. In the resulting *pyrG*-free strain, *akuA*Δ, we targeted the NRPS encoded by Aspbr1_34020, which based on the predictions above was the best candidate for malformin production. Six transformants were streak-purified and PCR analyzed, resulting in two homokaryotic deletion mutants of *mlfA* (see Fig. S3). Both strains were subsequently screened, alongside *akuA*Δ::AFL*pyrG* as reference, for their ability to produce malformin A2 and C after seven days of cultivation on yeast extract sucrose (YES) solid growth medium. The deletion of Aspbr1_34020 (*mlfA*Δ) showed a total abolishment of malformin production (Fig. 5a–c). Moreover, a genetic complementation by a constitutively expressed *mlfA* in the *mlfA*Δ strain re-established malformin production with the same adduct pattern as for the reference strain, thus confirming the role of *mlfA* in malformin A2 and C production (Fig. 5c,d).

## Discussion

With whole genus sequencing projects, research on natural products and the evolution of secondary metabolism experienced a paradigm shift due to the amount of generated data^10,12,47–49^. This study fills the gap for automated and scalable multispecies dereplication and classification of SMGCs using similarity networks.

Specific to our study is the mapping of phylogeny on SMGC families which then enables a relation of phylogeny to SMGC content and to chemical analyses. *A. niger* isolates which produce few different exometabolites^13^ show high, but not complete similarity of SMGC content. Species of distinct clades inside section *Nigri* can share 30–80% of SMGC depending on the distance of the species, showing a diversity similar to *Penicillium* clades as indicated recently^10^. Distantly related clades of biseriates and uniseriates inside section *Nigri* show SMGC similarity comparable to reference species comparisons. Thus, the SMGC diversity within the section has the same magnitude as within the whole genus *Aspergillus* — an observation hypothesized by analytical studies of produced metabolites in section *Nigri*^1^. On a genus level, the amount of shared SMGC families over large phylogenetic distances is higher in our study than estimated previously^19^.

As a result of our analysis, we were able to predict the malformin gene cluster in 18 strains and confirmed it in *A. brasiliensis*. Our results are in accordance with reports of producing strains as mentioned by Nielsen *et al*.^1^.

Identification of tailoring enzymes coding for disulphide-bond associated functions and establishment of a condensation domain model for the predicted gene cluster/synthetase helped us to further sustain our prediction. Upon deletion of *mlfA*, malformin production is abolished. Furthermore, we were able to show that complementation of *mlfA*Δ strains with *mlfA* can revive production of malformins. In combination, this makes us confident that *mlfA* is coding for a NRPS responsible for malformin production. We hypothesize the NRPS to act iteratively on one amino acid and possesses multiple amino acid specificities since multiple malformins disappear after deletion of the NRPS (Fig. 5).

Our study shows that SMGC similarity networks and families are ideal constructs for guilt by association based genetic dereplication and genome mining for SMs of interest. We were able to identify homologs of a gene cluster in 17 strains, which is silent in the original host under laboratory conditions^37^. Additionally, our method identified related pathways as e.g. trypacidin and secalonic acid and patulin, aculinic acid and yanuthone D. Hence, genetic dereplication uncovers new sets of SMGCs as targets for heterologous expression that might not be discovered by, e.g. OSMAC^50^ and facilitates further efforts to investigate the SMGCs of newly sequenced species.

Finally, similarity networks of SMGCs prove to serve for the genetic dereplication of SMGCs in several species and establish their phylogenetic distribution. Assessing and categorizing the metabolic potential of species in this automated manner will greatly facilitate the discovery of new relevant SMGCs.

## Materials and Methods

### Code availability

The code for the pipeline can be accessed under https://github.com/RoerdamAndersenLab/gene_cluster_networks_and_genetic_dereplication.

### Data collection

A customized version of SMURF^7^ was used to annotate secondary metabolite gene clusters throughout *Aspergillus* genomes (See details in^12^). Protein sequences, smurf annotations, interpro annotations and gff files were obtained from JGI (https://genome.jgi.doe.gov/).

### Creation of SMGC families

Families of gene clusters were created using the designed pipeline (Conceptual figure is shown in Fig. 1. The pipeline creates families of homologous gene clusters using local alignment of their protein sequences. It retains bidirectional hits that suffice the coverage cutoff and uses the percent identity to compute a similarity score for each query cluster to each hit cluster. Subsequently, the similarity scores are used to create a network of all SMGCs and random walk clustering is used to create families of SMGCs.

To run the pipeline, protein sequences, interpro, gff data and secondary metabolite gene cluster data were downloaded from JGI and loaded into a MySQL database. The pipeline can use this data from any source. All against all comparisons of all protein sequences in the set of genomes were created using BLAST+^22^ and subsetted for bidirectional hits between all secondary metabolite proteins using an E-value of 1e-10, at least 50% identity and a sum of coverage of 130% as cutoffs. These values were chosen to be relaxed in terms of identifying bidirectional hit. Subsequently, the identity values were aggregated from query to hit clusters to create a cluster similarity score,

$$\frac{sum(piden{t}_{tailoring})}{{n}_{tailoring}}\times 0.35+\frac{sum(piden{t}_{backbone})}{{n}_{backbone})}\times 0.65,$$

with *n* describing the maximum number of tailoring and backbone genes, respectively. The established connections were then used to create a network of secondary metabolite gene cluster proteins^51^ and random walk clustering^52^ in R^53^, with 1 step, was used to find families of related gene cluster proteins.

In the pipeline script (accessible from Github, see Code Availability below), the weights of the backbone versus tailoring enzymes can be changed. We examined weights from 50:50 to 0:100, but in our hands for this set of relatively closely related species, these weights performed the best in connecting clusters. These weights, connected clusters which varied in the number of tailoring enzymes, and connecting chemically related known compounds such as e.g. asperthecin and TAN-1612, see Results and Fig. 2 for details on families with multiple known compounds associated).

### Visualization of shared SMGC families

A heatmap containing hierarchically clustered column dendrograms was created using the heatmap.2 function of the gplots package^54^ with a matrix of percent shared SMGC as input. The column dendrogram is a result of the heatmap.2 function which creates a distance matrix of the input and clusters the result hierarchically using euclidean distance. A whole-genome phylogenetic tree was imposed on rows^55^.

### Mining for malformin producing NRPS

Created SMGC families were classified as potential producers of malformin according to three criteria. Strains which are known to produce malformin (*A. niger* CBS 513.88, *A. niger* NRRL3, *A. niger* ATCC 1015, *A. brasiliensis*, and *A. tubingensis*) should be included in the family; the clusters should include an NRPS of at least 12,000 nucleotides and tailoring enzymes should include the terms ‘glutathione’ or ‘disulphide’. From the two resulting families, the best hit was used for further investigation. Gene clusters were visualized using Gviz^56^.

### Whole genome phylogeny

A whole genome phylogenetic tree was generated to compare phylogeny to hirarchical clustering based on secondary metabolite family content. The phylogeny was constructed using 200 bidirectional best hits between species. These best hits were concatenated and aligned using MAFFT^57^ and conserved blocks extracted using Gblocks^58^. A maximum likelihood phylogeny was created using the trimmed alignments for multithreaded RAxML with PROTGAMMAWAG model and 100 bootstraps^59^.

### Prediction of condensation domain types

Condensation domains were extracted from protein sequences using annotations from InterproScan5^60^. Separated domains, i.e. domains smaller than 350 amino acids and less than 100 amino acids apart from a domain of the same type, were merged before proceeding. Resulting domain sequences were aligned using Clustal Omega^61^ and trimmed using trimal^62^ retaining sequences with over 65% residue coverage in over 80% of sequences and removing all columns with gaps in more than 20% of sequences with similarity lower than 0.001 but preserving at least 60% of columns. IQ-tree^63^ was used on aligned sequences using a LG + F + I + G4 substitution model^64^ and 1,000 times bootstrap^65^. Functions of condensation domains of the predicted malformin NRPS could be assigned by coclustering with known examples.

### Annotating SMGC families using MIBiG

The MIBiG database contains annotated gene clusters of the *Aspergillus* species used in this study (among many others) which made it a valuable resource for annotation of our data. To simplify the annotation process we chose to use local alignments of backbone proteins which, with conservative cutoffs, yield the original gene cluster from the MIBiG database in our data. Thus, gene cluster annotations were downloaded from the MIBiG database^21^ and 1,461 sequences of backbone proteins extracted using biopython^66^. Protein sequences were then blasted against our dataset. Hits reaching a percent identity, query coverage and hit coverage of over 95% were retained to find best hits in our dataset. Corresponding SMGC families were annotated as related cluster of the hit.

### Construction of mutant strains

The wild type culture (WT) *A. brasiliensis* (CBS 101740/IBT 21946)^67^ was used, to generate a uridine requiring *pyrG-* strain (*pyrG1*, BRA6), and from BRA6, a knockout strain of the Ku70 homolog *akuA* was created to enable efficient gene targeting^45^, see Table S1 for strains. Genomic DNA (gDNA) from WT *A. brasiliensis* was isolated via FastDNA SPIN Kit for Soil DNA extraction kit (MP Biomedicals, USA). All primers (Integrated DNA technologies) and plasmids are listed in Table S2 and Table S3, respectively. DNA fragments for USER cloning and plasmids were purified using illustra GFX PCR DNA and Gel Band Purification Kit (GE Healthcare Life Sciences) and GenElute Plasmid Miniprep Kit (Sigma-Aldrich), respectively, according to manufacturer’s instructions. Specifically, the sgRNAs for CRISPR/Cas9 plasmids targeting *A. brasiliensis pyrG* (Aspbr1_135933) and *akuA* (Aspbr1_0077313) were generated by amplifying two fragments of 545 bps and 424 bps, respectively, using template pFC334^46^ and primers P1 + P5 and P2 + P6 (*pyrG*) and P3 + P5 and P4 + P6 (*akuA*). In both cases, the two fragments were USER-cloned into pFC332^46^, and the resulting plasmids were verified by enzymatic digestion with BspEI according to manufacturer’s instructions (New England Biolabs, NEB), and sequencing of the sgRNA part (StarSEQ). PCR conditions for cloning-fragment amplification and USER-cloning procedure were as described in^68^. USER cassettes were based on PacI/Nt.BbvCI sites. The principle and procedure for assembly of CRISPR/Cas9 mediated gene editing were as according to^46^. For deleting *akuA* and *mlfA* (Aspbr1_34020), up- and downstream sequences flanking the coding sequences of the genes were amplified by PCR and USER cloned into the gene-targeting vector pFC478 that employs *pyrG* from *A. flavus* flanked by a direct repeat sequence^46^. For *akuA*, 2266 bps of *akuA*-Up and 2284 bps of *akuA*-Down primers P7 + P8 and P9 + P10 were used, respectively. Correspondingly, primers P11 + P12 and P13 + P14 amplified 2192 bps and 1842 bps flanking *mlfA*. Gene targeting plasmids were linearized and verified by enzymatic digestion with SwaI, according to manufacturer’s instructions (NEB). The *akuA* gene-targeting construct was co-transformed with circular *akuA* targeting CRISPR/Cas9 plasmid into BRA6 selecting for only *pyrG*. Homokaryosis and deletion of *akuA*, as well as the subsequent *pyrG* marker loss after counter-selection on MM + 5-FOA was verified by diagnostic tissue-PCR (P15-P18) as described in^46^ and Fig. S3. Specifically, for the *akuA*Δ strain, BRA9, *pyrG* was excised resulting in strain BRA10 (*pyrG1*, *akuA*Δ), which was applied as background for the deletion of *mlfA*. Tissue-PCR using primers P19-P24 verified the deletion of *mlfA* (BRA30 and BRA52, see Fig. S3). For complementation of the *mlfA* deletion, see Table S2 and Fig. S3. Protoplastation and transformation of BRA1, BRA6, BRA10, and BRA52 were and conducted as described in^69^ and^46^, respectively. All *A. brasiliensis* strains were cultivated at 30 °C on minimal medium (MM), supplemented with 10 mM uridine if required for growth. The MM, transformation media (TM) and media for *pyrG* counter-selection (MM + 5-FOA) were prepared as described in^46^. All transformations employing CRISPR/Cas9 vectors used hygromycin B (100 *μ*g/ml, Invivogen) for selection. Yeast extract sucrose (YES^70^) growth media was used for chemical analysis. Chemical competent *Escherichia coli* DH5*α* were applied for vector assembly and plasmid propagation at 37 °C, and *E. coli* cultivations were carried out in Lumia Broth (LB) media (1% Bacto tryptone, 0.5% Bacto yeast extract, 1% NaCl, pH 7.0) supplemented with 0.1% ampicillin. All solid media were supplied with 2% agar.

### Secondary metabolite extraction and analysis

Extraction of secondary metabolites from solid media (CYA and YES) 6 plugs (6 mm) were based on samples across the radius of the fungal colony, transferred to a microcentrifuge tube and covered in ethyl acetate/2-propanol 3:1(v/v) with 1% (v/v) formic acid for 60 min ultrasonication. The extraction solvent was transferred to a clean vial, solvents evaporated using N_2_ flow, and the residues on the tube walls were re-dissolved in methanol for 30 min by ultrasonication. The samples were centrifuged at 15,000 g and the supernatant transferred to a HPLC auto sampler vial. UHPLC-DAD-QTOFMS was performed on an Agilent Infinity 1290 UHPLC system equipped with a diode array detector. Separation was done on a 250 × 2.1 mm i.d., 2.7 *μ*m, Poroshell 120 Phenyl Hexyl column (Agilent Technologies, Santa Clara, CA) held at 60 C. Subsamples of 1 *μ*L, were eluted with a flow rate of 0.35 mL/min using A: water with 20 mM formic acid and B: acetonitrile with 20 mM formic acid as a gradient system starting at 90% A, which linearly dropped to 10% in 15 min, and held for 2 min before returning to 90% for 2 min. Acetonitrile, methanol, ethyl acetate, 2-propanol and formic acid were analytical grade (Sigma-Aldrich, St. Louis, MO, USA). Water, acetonitrile and formic acid for MS solvents were all LC-MS grade (Sigma-Aldrich). Mass spectrometry (MS) detection was performed on an Agilent 6545 QTOF MS equipped with an Agilent dual jet stream ESI operated in ESI+ mode, with MS spectra recorded as centroid data, at an m/z of 100 to 1,700, and auto MS/HRMS fragmentation was performed at three collision energies (10, 20, 40 eV), on the three most intense precursor peaks per cycle. The acquisition was 10 spectra/s. Data were treated in Agilent MassHunter Qualitative Analysis, and compounds were detected using extracted ion chromatograms (EICs) ± m/z 0.005 Da of the theoretical masses^71^. MSHRMS were evaluated against a database of 1,500 compounds, while HRMS and MS/HRMS peaks were matched against around 3,000 known and suspected *Aspergillus* compounds. Reference standards of malformins C and A were co-analysed in the sequence. Malformin A2 (C_22_H_37_O_5_N_5_S_2_) and C (C_23_H_39_O_5_N_5_S_2_, Fig. 5) were detected at using EICs of expected adducts ([M + H]^+^, [M + NH_4_]^+^, [M + Na]^+^) based on the calculated monoisotopic mass [M], 515.2236 Da and 529.2393 Da.

## Electronic supplementary material

Supplementary information

Dataset 1

## Data Availability

Data used to generate results of this study can be found under: https://files.dtu.dk/u/tdYsymlWLM2n1izL/gene_cluster_networks_and_genetic_dereplication?l. Fungal genomes are deposited at jgi https://genome.jgi.doe.gov/.
