# Supplementary material for: Uncovering secondary metabolite evolution and biosynthesis using gene cluster networks and genetic dereplication
Source: Sci Rep. 2018 Dec 18;8:17957. doi: 10.1038/s41598-018-36561-3 (PMC6298953; doi:10.1038/s41598-018-36561-3)
Supplement: Supplementary file 1 — Supplementary information [file 41598_2018_36561_MOESM1_ESM.pdf]

Uncovering secondary metabolite evolution and biosynthesis using gene cluster networks and genetic dereplication

Sebastian Theobald[1,4], Tammi Camilla Vesth[1], Jakob Kræmmer Rendsvig[1], Kristian Fog Nielsen[1,5], Robert Riley[2,6], Lucas Magalhães de Abreu[3], Asaf Salamov[2], Jens Christian Frisvad[1], Thomas Ostenfeld Larsen[1], Mikael Rørdam Andersen[1,*], and Jakob Blæsbjerg Hoof[1,*]

[1]Department of Biotechnology and Biomedicine, Technical University of Denmark, DK-2800 Kongens Lyngby, Denmark

[2]Department of Energy Joint Genome Institute, Walnut Creek, CA, USA

[3]Department of Plant Pathology, Federal University of Viçosa, Viçosa, Brasil

[4]Current Address: The Novo Nordisk Foundation for Biosustainability, Technical University of Denmark, DK-2800 Kgs. Lyngby, Denmark

[5]Current Address: Chr. Hansen Holding A/S, DK-2970 Hoersholm, Denmark

[6]Current Address: Amyris, Inc., Emeryville, CA, USA.

[*]These authors contributed equally to this work; M.R.A. (E-mail: mr@bio.dtu.dk) is corresponding author on the computational analysis and J.B.H. is corresponding author on the experimental work (jblni@dtu.dk)

Supplementary Information (SI)

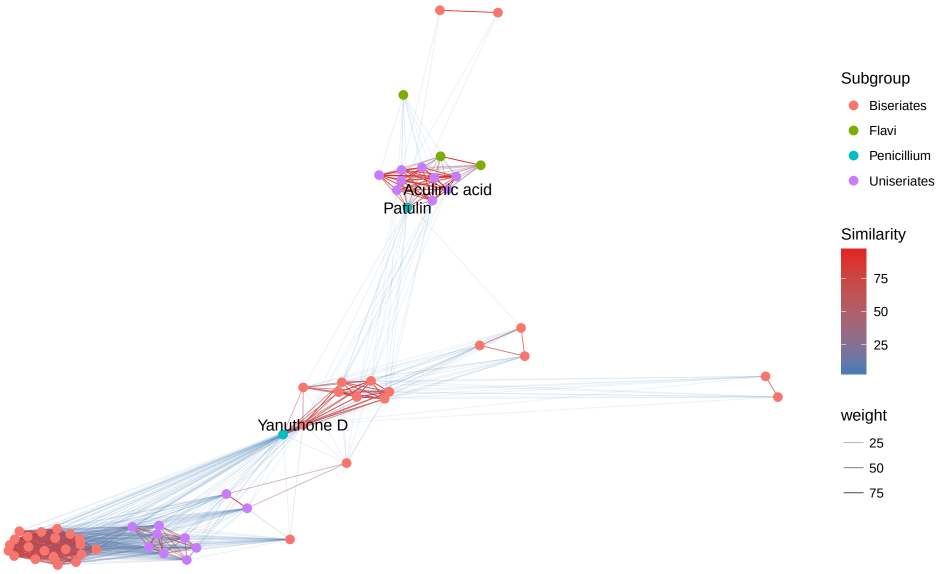

Figure 1. Network of cluster similarities with all gene clusters connected to the patulin gene cluster. Similarity is indicated by edge color, clade by node color and annotated family by shape. The network shows four gene cluster families associated with the patulin gene cluster.

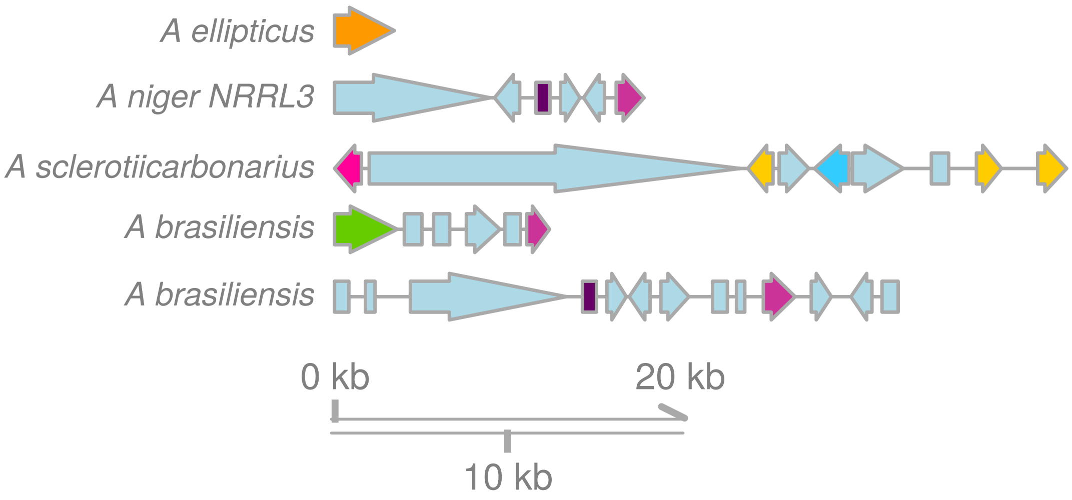

Figure 2. Gene clusters which have been removed from predicted malformin gene cluster family. The gene clusters shown in this figure were only assigned to the family by greedy prediction or similarity to irrelevant parts of the target malformin gene cluster.

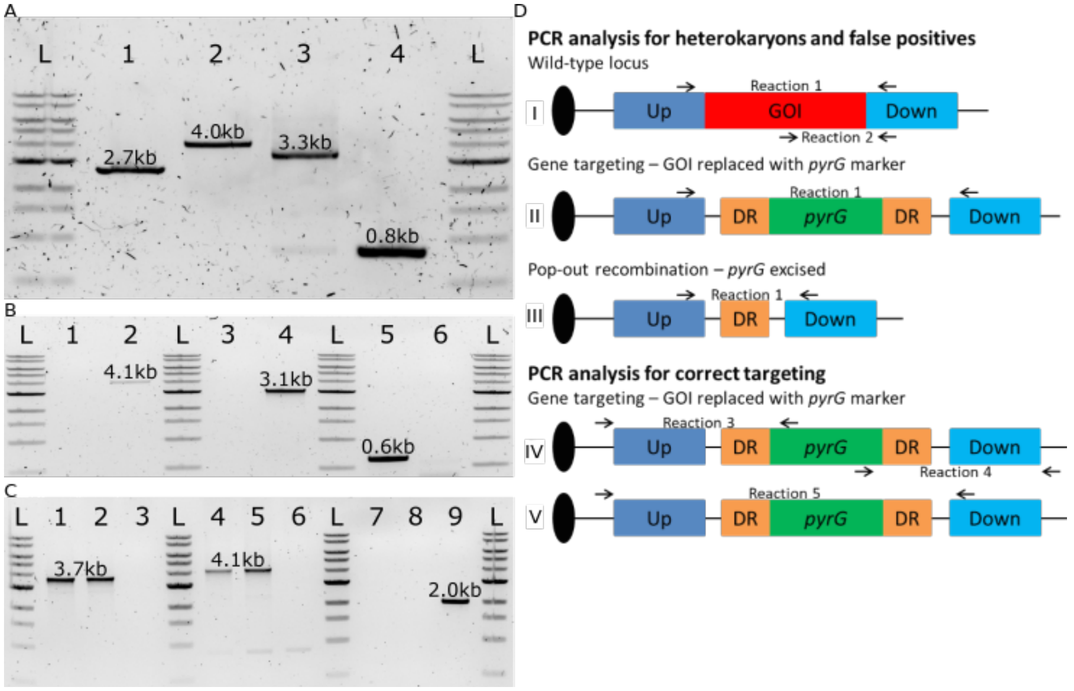

Figure 3. A *akuA* Δ strain verification. The figure shows the PCR verification of the *akuA* (Aspbr1_0077313) deletion strain. In each lane (1-4) contains expected approximate band length, and sizes are compared to 1 kb ladder (L). Lane 1 shows reaction 1 (**D**) with primers P15+P16 (Table S2) on the wild-type strain, producing a band of 2.7 kb. After transformation of the *pyrG1* strain with *akuA* gene-targeting DNA construct, the correct integration at the *akuA* locus was tested by reaction 3 (primers P17+P18), yielding a band of 4.0 kb (lane 2), verifying the integration of the AFL*pyrG* marker into the *akuA* locus, thus, this strain is *akuA*Δ::AFL*pyrG*. Lane 3 and 4 show reaction 1 respectively on *akuA*Δ::AFL*pyrG* (3.3 kb) and the *akuA*Δ strain (0.8 kb) created after pop-out recombination of AFL*pyrG*. The marker excision band (0.8 kb) and a product of approximately 6 kb are noticeable in lane 3, which we ascribe to PCR products formed from a direct repeat annealing during PCR.
B *mlfA*Δ strain verification. The figure shows an example of the PCR verification for one of the *mlfA* (Aspbr1_34020) deletion strains (BRA30, see Table S1). The agarose gel is divided into three sections, each showing two lanes representing a specific reaction on first the wild-type strain (lanes 1, 3, 5) and secondly the *mlfA*Δ::AFL*pyrG* (lanes 2, 4, 6). The four L lanes show the 1 kb ladder. The expected approximate band length is added to lanes 2, 4 and 5, whereas in lanes 1, 3 and 6 no band is expected. The first two sections show the outcome from reaction 3 (primers P19+P24) and 4 (primers P22+P23), respectively. Lanes 1 and 3 represent PCR on the wild-type strain where AFL*pyrG* is not present in the *mlfA* locus, thus no bands are seen. Reactions on the *mlfA*Δ::AFL*pyrG* mutant (lanes 2+4) show the expected products of 4.1 kb and 3.1 kb, respectively, verifying correct targeted integration. The third and last section with lanes 5 and 6 revealed by reaction 2 (primers P20+P21) that *mlfA* was only present in wild-type strain (lane 5) but not in *mlfA*Δ::AFL*pyrG* (lane 6), where two faint unspecific smaller products was observed.
C Verification of *mlfA*-Oex strains. Lanes 1-3 and lanes 4-6 respectively show the results by agarose gel electrophoresis after PCR reaction 3 and 4 (primers P27+P28 and P29+P30), with the internal primers binding in the integrated *mlfA* instead of the *pyrG* marker. The *mlfA*-Oex strains gave the expected bands for both reaction 3 (lane 1+2; 3.7 kb) and reaction 4 (lane 4+5; 4.1 kb) confirming targeted integration of the cassette for both ends at the integration site (IS1) locus. Wild-type controls did not show any product (lane 3 and 6). Testing for presence of untransformed wild-type nuclei was accomplished by reaction 5 (primers P25+P26) in lanes 7-9, where no band is seen for the *mlfA*-Oex strains (lanes 7+8; expected band length 22kb) and a 2.0 kb band for the wild-type control (lane 9), confirming both strains to be homokaryotic mutants in the IS1 locus.
D Setup for verification of mutant strains. The PCR strategy employed ensures verification of two aspects. Firstly, the deletion cassettes with the *pyrG* marker has been correctly integrated in the targeted locus, gene of interest (GOI), and secondly, no untransformed wild-type nuclei are present in the mutant strains. All the mutant strains analyzed were compared to reference gDNA. Specifically, reaction 1 amplifies the target coding sequence from primers placed into the proximal targeting sequences (Up and Down) applied for homologous recombination. The main application of reaction 1 is to show if the strain is heterokaryon (has wild-type nuclei), **I**, while it also indicates whether the marker has replaced the target, **II**. Moreover, it is efficient to show whether the *pyrG* marker is excised after counter-selection on 5-FOA, **III**. Reaction 2 is used for very large stretches of DNA, **I**, that are difficult to amplify in reaction 1, as in the case of *mlfA*. Reactions 3 and 4 validate correct targeting by employing primers binding in unique locations outside the targeting sequences that each form a pair with primers internally in the marker gene, *IV*. Reaction 5 may likewise validate correct targeting by the one primer binding in unique location outside the targeting sequence, while also determining if the strain is heterokaryon as it provides a band even for wild-type strains since the second primer is located in the targeting sequence instead of the marker gene, **V**.

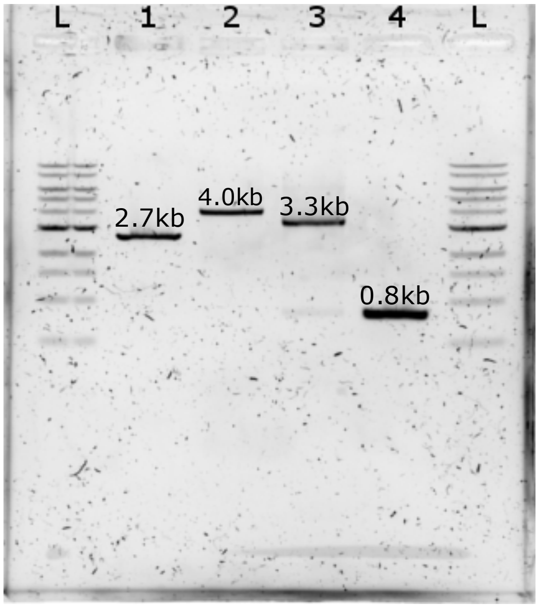

Figure 4. Original image for Fig.3 A. For description see caption of Fig. 3

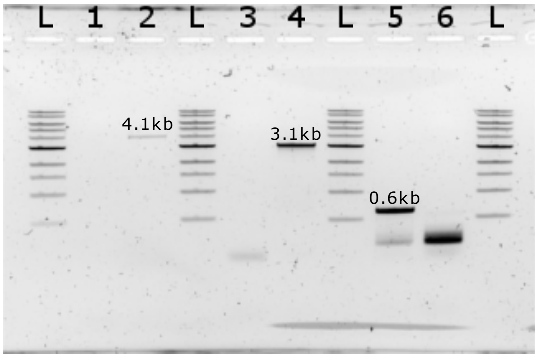

Figure 5. Original image for Fig.3 B. For description see caption of Fig. 3

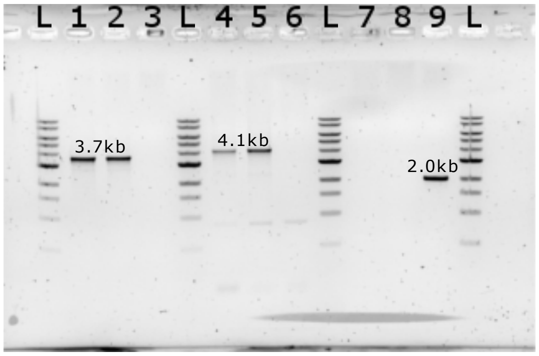

Figure 6. Original image for Fig.3 C. For description see caption of Fig. 3

Table 1. Strains of *Aspergillus brasiliensis* CBS 101740 used in this study

Strain ID	Genotype	Source
BRA1	Wild-type	[67]
BRA6	*pyrG1*	This study
BRA9	*pyrG1*, *akuA*Δ::AFL*pyrG*	This study
BRA10	*pyrG1*, *akuA*Δ	This study
BRA30	*pyrG1*, *akuA*Δ, *mlfA*Δ::AFL*pyrG*	This study
BRA44	*pyrG1*, *akuA*Δ, *mlfA*Δ::AFL*pyrG*	This study
BRA52	*pyrG1*, *akuA*Δ, *mlfA*Δ	This study
BRA67	*pyrG1*, *akuA*Δ, *mlfA*Δ, IS1::*PgpdA*-*mlfA*-*TtrpC*-DR-AFUM*pyrG*-DR	This study
BRA68	*pyrG1*, *akuA*Δ, *mlfA*Δ, IS1::*PgpdA*-*mlfA*-*TtrpC*-DR-AFUM*pyrG*-DR	This study

Nomenclature for the TSs refers to the numbered species in the table. AFUM: *Aspergillus fumigatus*, AFL: *A. flavus*

Table 2. List of primers used in this study

Code	Name	Sequence (5'-3')
P1	gRNA-ABRApyrG-rv	AGCTTACUCGTTTCGTCCTCACGGACTCATCA-GGCCTCTCGGTGATGTCTGCTCAAGCG
P2	gRNA-ABRApyrG-fwd	AGTAAGCUCGTCGCCTCTTGGCAAGAGCATTGG-TTTTAGAGCTAGAAATAGCAAGTTAAA
P3	gRNA-ABRAakuA-rv	AGCTTACUCGTTTCGTCCTCACGGACTCATCAG-GATGAACGGTGATGTCTGCTCAAGCG
P4	gRNA-ABRAakuA-fwd	AGTAAGCUCGTCGATGAAGATGAAGACAGTCG-GTTTTAGAGCTAGAAATAGCAAGTTAAA
P5	PgpdA-pac-up-fwd	GGGTTTAAUGCGTAAGCTCCCTAATTGGC
P6	TtrpC-short-pac-dw-rv	GGTCTTAAUGAGCCAAGAGCGGATTCCTC
P7	ABRAakuA-Dl-Up-FU	GGGTTTAAUGACTGGTGAGTGATTGTGGGAG
P8	ABRAakuA-Dl-Up-RU	GGACTTAAUGTGGGGCTGCTGTGTCTG
P9	ABRAakuA-Dl-Dw-FU	GGCATTAAUGCATAATAGGATGGTCGGGTTCG
P10	ABRAakuA-Dl-Dw-RU	GGTCTTAAUGCCTTGGTAGGCAGACGAATAG
P11	ABRA34020-Dl-Up-FU	GGGTTTAAUGAGGGAGTGAAAGTCGTCG
P12	ABRA34020-Dl-Up-RU	GGACTTAAUGAAGGAGGTGAAGTTACTAGGAC
P13	ABRA34020-Dl-Dw-FU	GGCATTAAUGTGGTTGCCATGAGTGAAAGTG
P14	ABRA34020-Dl-Dw-RU	GGTCTTAAUGATGCAGTGCAGGTTGGAG
P15	ABRAakuA-ChkGap-F	CCAGCCAGCGTCATCAATTAC
P16	ABRAakuA-ChkGap-R	CCTACCCCGACATCCAACC
P17	ABRAakuA-ChkUP-F	CCTTCCCAGCTCTCAAGTCC
P18	AFLpyrG-Chk-int-R2	CTAGATCACATGTAAGTGGCATCCC
P19	ABRA34020-Chk-Up-F	CCGTCCGAAGATCAATCCGAC
P20	ABRA34020-Chk-Gap-F	GCACGTCGGCTGTGATGG
P21	ABRA34020-Chk-Int5'-R	GAGTGTGATCTGGATTCGGG
P22	ABRA-34020-ChkDw-R	CCCACCATTTGTAACGCACTG
P23	AFLpyrG-Chk-Dw-F	CCCACCACCCCCTACTCTAACAC
P24	AFLpyrG-Int-R-BP	CCCATCACAAACTTCTTATACTTCCGA
P25	ABIS3-ChkDw-R	CACCACAGCCATCAAATCC
P26	ABIS3-ChkGap-F	CGACAACCCCAACAACG
P27	ABIS3-Chk-Up-F2	GGTGTCTGTGTGTGACAGACTAG
P28	ABRA34020-Chk-Int5'-R	GAGTGTGATCTGGATTCGGG
P29	ABIS3-Chk-Dw-R2	GTGTCTTTGGTACCATGGGAGAG
P30	ABRA34020-GapChk-F	GAAGTGTGGGGTTGATGTG
P31	ABRA-IS-Up-FU2	GGGTTTAAUGGCTTCTCCATCCTTCTACATG
P32	ABRA-IS-Up-RU2	AGGGAAUTGTAGGTAAACCAGCACGCTC
P33	ABRA-IS-Dw-FU	GGCATTAAUGATGAGCATATTGTGTGAGG
P34	ABRA-IS-Dw-RU	GGTCTTAAUGGGATGGGTATGTTGGA
P35	PgpdA(2.3kb)-FU-ANIS5-TSI	ATTCCCUTGTATCTCTACACACAGGC
P36	PgpdA-Rev-RU PacI.Reg	AAACCCUCAGCGCGGTAGTGATGTCTGCTCAAG
P37	TtrpC-PacI.Reg-FU	AGGGTTUAATTAAGACCTCAGCGGATCCACTTAACGTTAC
P38	TtrpC-RU-PacI	GGACTTAAUCGCTTACACAGTACACGAG
P39	ABRA34020-F1-FU-PacI 2	GGGTTTAAUATGAGTCGCTTTTCCTGC
P40	ABRA34020-F1-RU 2	AGCCCCTGUCCGTGTGTT
P41	ABRA34020-F2-FU	ACAGGGGCUGTTCAACACG
P42	ABRA34020-F2-RU	AGCGAGAUGCGGACAGG
P43	ABRA34020-F3-FU 2	ATCTCGCUCGGTGACCAAG
P44	ABRA34020-F3-RU 2	ACTCCGUCAACTGGAACATCTC
P45	ABRA34020-F4-FU 2	ACGGAGUGGAGATGATGGTC
P46	ABRA34020-F4-RU-PacI	GGTCTTAAUTCAAACACAGACACCCCGAG

P1-6 CRISPR/*cas9* construction, P7-14 deletion plasmid construction, P15-30 strain verification. P31-46 Integration site (IS) and *mlfA* expression vector construction as in [68]. Assembly of *mlfA* was performed in four fragments.

Table 3. List of plasmids used in this study

Plasmid ID	Application	Content
pFC334 [46]	CRISPR sgRNA template	*argB*, *Ptef1*-*cas9*-*Ttef1*, *PgpdA*-sgRNA-*TtrpC*
pFC332 [46]	CRISPR/*cas9* vector	*hygR*, *Ptef1*-*cas9*-*Ttef1*
pFC837	CRISPR/*cas9 pyrG*[b]	*hygR*, *Ptef1*-*cas9*-*Ttef1*, *PgpdA*-sgRNA(*pyrG*)-*TtrpC*
pFC715	CRISPR/*cas9 akuA*[c]	*hygR*, *Ptef1*-*cas9*-*Ttef1*, *PgpdA*-sgRNA(*akuA*)-*TtrpC*
pFC478 [46]	Gene deletion vector	DR-AFL*pyrG*-DR
pFC645	Deletion plasmid *akuA*	Up_{akuA}-DR-AFL*pyrG*-DR-$Down_{akuA}$
pFC809	Deletion plasmid *mlfA*	Up_{mlfA}-DR-AFL*pyrG*-DR-$Down_{mlfA}$
pFC3 [45]	Base overexpression vector	DR-AFUM*pyrG*-DR
pFC1116	*mlfA* overexpression	Up_{IS1}-*PgpdA*-*mlfA*-*TtrpC*-DR-AFUM*pyrG*-DR-$Down_{IS1}$

b: Protospacer for targeting *A. brasiliensis pyrG* is GCCTCTTGGCAAGAGCATTG, c: Protospacer for targeting *A. brasiliensis akuA* is GATGAAGATGAAGACAGTCG. All plasmids contain the *ampR* and *AMA1* sequence for propagation in *E. coli* and Aspergilli, respectively.
